# Potential roles of DNA methylation in the initiation and establishment of replicative senescence revealed by array-based methylome and transcriptome analyses

**DOI:** 10.1371/journal.pone.0171431

**Published:** 2017-02-03

**Authors:** Mizuho Sakaki, Yukiko Ebihara, Kohji Okamura, Kazuhiko Nakabayashi, Arisa Igarashi, Kenji Matsumoto, Kenichiro Hata, Yoshiro Kobayashi, Kayoko Maehara

**Affiliations:** 1 Department of Maternal-Fetal Biology, National Research Institute for Child Health and Development, Setagaya, Tokyo, Japan; 2 Department of Biomolecular Science, Graduate School of Science, Toho University, Funabashi, Chiba, Japan; 3 Department of Systems BioMedicine, National Research Institute for Child Health and Development, Setagaya, Tokyo, Japan; 4 Department of Allergy and Clinical Immunology, National Research Institute for Child Health and Development, Setagaya, Tokyo, Japan; 5 Department of Nutrition, Graduate School of Health Science, Kio University, Kitakatsuragi, Nara, Japan; Centre de Recherche en Cancerologie de Lyon, FRANCE

## Abstract

Cellular senescence is classified into two groups: replicative and premature senescence. Gene expression and epigenetic changes are reported to differ between these two groups and cell types. Normal human diploid fibroblast TIG-3 cells have often been used in cellular senescence research; however, their epigenetic profiles are still not fully understood. To elucidate how cellular senescence is epigenetically regulated in TIG-3 cells, we analyzed the gene expression and DNA methylation profiles of three types of senescent cells, namely, replicatively senescent, *ras*-induced senescent (RIS), and non-permissive temperature-induced senescent SVts8 cells, using gene expression and DNA methylation microarrays. The expression of genes involved in the cell cycle and immune response was commonly either down- or up-regulated in the three types of senescent cells, respectively. The altered DNA methylation patterns were observed in replicatively senescent cells, but not in prematurely senescent cells. Interestingly, hypomethylated CpG sites detected on non-CpG island regions (“open sea”) were enriched in immune response-related genes that had non-CpG island promoters. The integrated analysis of gene expression and methylation in replicatively senescent cells demonstrated that differentially expressed 867 genes, including cell cycle- and immune response-related genes, were associated with DNA methylation changes in CpG sites close to the transcription start sites (TSSs). Furthermore, several miRNAs regulated in part through DNA methylation were found to affect the expression of their targeted genes. Taken together, these results indicate that the epigenetic changes of DNA methylation regulate the expression of a certain portion of genes and partly contribute to the introduction and establishment of replicative senescence.

## Introduction

Cellular senescence is the irreversible cessation of cell proliferation [1] and is classified into two groups: replicative senescence and premature senescence [2]. Replicative senescence is caused by telomere shortening due to repeated DNA replication [3], while premature senescence is caused by stress, such as oncogene activation [4] and reactive oxygen species (ROS) [5], without apparent loss of telomere length and function. Although cellular senescence has been shown to be associated with tumor suppression in several cancers [2, 6, 7], it has been reportedly involved in cancer progression through the induction of epithelial-mesenchymal transitions and tumor invasion [8]. In addition, senescent cells secrete several factors associated with inflammation such as interleukin (IL)-6 and IL-8 [9], which are referred to as senescence-associated secretory phenotypes (SASP). Recently, SASP has been implicated in the pathogenesis of age-related diseases such as rheumatoid arthritis, periodontitis and Alzheimer’s disease [8, 10]. Therefore, elucidation of the mechanism contributing to induction and establishment of the senescent state will help overcome such age-related diseases.

Epigenetic regulation such as histone modification, DNA methylation and interference with micro RNA (miRNA) is one of the mechanisms that modulate gene expression. Alteration of the chromatin structure occurs in human senescent fibroblasts, where epigenetic regulation contributes to the establishment of the senescent state partly through the p16^INK4A^ / retinoblastoma (RB) protein pathway [11]. Modification of histones, trimethylated histone H3 at lysine9 (H3K9me3) and trimethylated histone H3 at lysine 27 (H3K27me3) is enriched in the senescent-associated heterochromatic foci (SAHF), although the spreading of repressive histone marks is not necessary for SAHF formation [12]. So far as known, histone modification is reportedly involved in expression of key molecules of senescence, such as p16^INK4A^, p14^ARF^ and p53. Loss of H3K27me3 is involved in expression of p16^INK4A^ and p14^ARF^, whereas H4 acetylation and trimethylation of histone H3 at lysine 4 (H3K4me3) are involved in expression of p53 [13–16]. Furthermore, Takahashi, A. *et al*. [17] showed that the expression levels of SASP factors, IL-6 and IL-8, are regulated by demethylation of H3K9 through the APC/C^Cdh1^-G9a/GLP pathway.

DNA methylation changes have also been observed during cellular senescence and individual aging. Many studies have elucidated the role of epigenetics in senescence using various approaches such as pyrosequencing, array-based methylome and combined bisulfite restriction analysis (COBRA), as well as using various types of cells and different tissues derived from genetically different individuals [18–21]. These studies have shown that DNA methylation profiles are tissue- and cell-type specific, and the epigenetic control of gene expression seems to promote in part tissue- and cell-type specific differentiation in addition to cellular senescence. For instance, the DNA methylation profile of fibroblast cells is different to that of mesenchymal stromal cells [22]. Moreover, principal component analysis showed that the methylation pattern of fibroblast cells from one dermal region is different to that from other dermal regions [23]. Christensen *et al*. also showed interindividual variation in methylation profiles among 11 tissues, including blood and brain [24]. In contrast to the studies using genetically different tissues and cells derived from individuals, age-related changes in DNA methylation between monozygotic twins were reported to arise with chronological time, indicating that the differences in genetically identical individuals are driven by different cellular responses to environmental changes [25]. Therefore, we hypothesized that a specific type of cultured cell leading to senescent states induced by different methods and demonstrating differences in methylation profiles will allow us to characterize in detail the role of epigenetic regulation in response to cellular senescence.

The effects of DNA methylation on gene transcription have been extensively studied in relation to the states of CpG islands (CGIs) near the transcription start sites (TSS). According to recent studies examining the relationship between gene body methylation and gene expression, hypermethylation correlated to not only high gene expression but also low gene expression [26], suggesting a more complex regulation. Varley *et*. *al*. also reported that the relationship between methylation and gene expression is context-dependent, although the current models reported by several groups indicate that methylation in the promoter regions is associated with gene silencing, and gene body methylation is associated with expression [20].

Furthermore, some senescence-associated genes are regulated in part by miRNA [27–31]. MiR-34 has been well known as a tumor suppressor and its targeted genes encode the cell cycle regulators including E2F, c-Myc, cyclin D1, cyclin E2, cdk4, and cdk6 [32–34]. Recent studies on cancers have revealed that the expression of some tumor suppressive miRNAs is regulated by DNA methylation in the miRNA promoter regions [35–37]. Despite these recent advances, much of the relationship between DNA methylation and miRNA expression and how this relates to senescence genes remains unclear.

In this study, we examined characteristic features of DNA methylation during cellular senescence using TIG-3 cells established from fetal lung fibroblasts. Although such TIG-3 cells have been a common focus in senescence research, much of the epigenetics remains unexplored. To test the hypothesis that the differences in genetically identical cells are driven by different cellular responses to senescence, we examined array-based gene expression and DNA methylation profiles using genetically identical TIG-3 cells which had been induced to a senescent state by three different methods, namely, replicatively senescent, *ras*-induced senescent (RIS), and senescent SVts8 cells. Originally derived from TIG-3 cells, SVts8 cells can induce senescence under non-permissive temperature by inactivation of the temperature-sensitive SV40 large T antigen. We then searched for the positional trend of methylation changes and the possible effects of DNA methylation changes on gene expression using integrated analysis.

## Materials and methods

### Cells and cell culture

Normal human diploid fibroblast TIG-3 cells (obtained from the Health Science Research Resources Bank, Japan) were cultured in DMEM+GlutaMAX-I (GIBCO) supplemented with 10% fetal bovine serum (FBS) (HyClone) and 1% penicillin/streptomycin (Nacalai Tesque) at 37°C under a 5% CO_2_ atmosphere. TIG-3 cells were cultured until they senesced at population doubling level (PDL) 85. The TIG-3 cells were harvested for DNA extraction at PDL 36, 49, 69 and 85, for RNA extraction to analyze gene expression at PDL 36 and 84, and for RNA extraction to analyze miRNA expression at PDL 44, 60, 78 and 80.

To prepare RIS cells, retroviral infection was performed as reported previously [38]. Briefly, Phoenix-Eco cells (obtained from Dr. G. P. Nolan, Stanford University, CA, USA) were transfected with the pBabe-puro-H-Ras-V12 or pBabe-puro plasmid by the Chen-Okayama method [39]. Viral supernatants were prepared from the cells after transfection, passed through a 0.45-μm-pore-size syringe filter, and pooled. The supernatant and 8 μg/mL hexadimethrine bromide (Sigma-Aldrich) were added to TIG-3 cells expressing an ecotropic receptor at a proliferating phase. After infection, the cells were selected with growth medium containing 300 μg/mL G418 and 2 μg/mL puromycin for 9 days before being harvested.

SVts8 cells (obtained from the Health Science Research Resources Bank, Japan) [40] continued to proliferate at a permissive temperature (33.5°C), because of suppression of RB and p53 through induction of a temperature-sensitive mutant of the simian virus (SV) 40 large T antigen to TIG-3 cells and the high ability of telomere maintenance. Senescent SVts8 cells were obtained by culturing SVts8 cells at a non-permissive temperature (38°C) for 6 days.

### Microarray assays

#### Gene expression

Total RNA was isolated with a ReliaPrep RNA Cell MiniPrep System (Promega) according to the manufacturer’s instructions. Starting with 200 ng of the isolated RNA for each sample, double stranded cDNA and cyanine 3 labeled cRNA were synthesized using a low input quick amp labeling kit (one-color) and RNA spike-in kit (Agilent). The labeled cRNA was purified with an RNeasy mini kit (Qiagen), and hybridized to a SurePrint G3 Human GE microarray 8×60K Ver. 2.0 (Agilent). After washing the microarray to remove unhybridized cRNA, the microarray was scanned with an Agilent DNA microarray scanner G2505B, and then feature extraction was performed using the GE1_QCMT_Sep09 protocol.

#### DNA methylation

Genomic DNA was isolated using DNeasy Blood & Tissue (Qiagen) according to the manufacturer’s instructions. Genomic DNA (1.5 μg) was bisulfite-converted using the EpiTec Plus DNA Bisulfite Kit (Qiagen). From each sample, 300 ng of bisulfite-treated DNA was subjected to DNA methylation profiling using an Infinium HumanMethylation27 or HumanMethylation450 BeadChip array (Illumina) according to the manufacturer’s standard protocol. The array slides were scanned with an iScan system (Illumina).

#### MicroRNA (miRNA) expression

Total RNA including miRNA was isolated with a ReliaPrep miRNA Cell and Tissue MiniPrep System (Promega) according to the manufacturer’s instructions. Approximately 100 ng of the isolated RNA for each sample was labeled with cyanine 3 using miRNA Complete Labelling and Hyb Kit (Agilent) according to the manufacturer’s protocols. The labeled RNA was hybridized to a SurePrint G3 Human miRNA Microarray (Release 21.0) (Agilent). After washing, the microarray was immediately scanned using one color scan setting for 8×60k array slides with an Agilent DNA microarray scanner G2505C. The images were extracted with Feature Extraction Software 10.7.3.1 (Agilent) using default parameters.

### Data analysis

#### Gene expression

The SurePrint G3 Human GE microarray data were analyzed using the Subio Platform (Ver. 1.18.4625). The data were normalized as to low signal cutoff (cutoff 1.0 and replace), log transformation (base 2), and global normalization (percentile 75), and then the ratios to those of the control sample (mean) were obtained. In this study, > = 2-fold and = < 0.5-fold changes were regarded as up- and down-regulated gene expression, respectively. In order to calculate fold-change differences and construct a heatmap, each gene expression level in the senescent cells was compared with that in the control cells, namely replicative senescent cells versus proliferating cells, RIS cells versus cells infected with the empty vector, and senescent SVts8 cells versus proliferating SVts8 cells. The heatmap with sample clustering was drawn using Subio Platform with Uncentered Correlation. The microarray data for gene expression have been deposited in the GEO (GSE81798).

#### DNA methylation

The Infinium HumanMethylation27 BeadChip includes probes for 27,578 CpG sites [41]. The Infinium HumanMethylation450 BeadChip includes probes for 485,577 CpG sites covering 21,231 RefSeq genes (99%), and 26,658 CGIs (96%), and 3,091 probes for non-CpG loci [42]. The image data obtained by the iScan system were subjected to background subtraction and control normalization using GenomeStudio V2011.1 (Illumina). Methylation levels were calculated as β values (= intensity of the methylation allele / [intensity of the unmethylated allele + intensity of the methylated allele +100]), which ranged from 0 (0% methylation) to 1 (100% methylation). Probes for CpG sites with a detection p-value of > 0.05 or a blank β value were eliminated from further analysis. The β values of control cells were subtracted from those of senescent cells (Δβ). Δβ > = 0.2 and Δβ = < −0.2 were regarded as hyper- and hypomethylation in this study. TIG-3 cells at PDL 36 were regarded as control cells for those at higher PDLs (49, 69, and 85). An Infinium HumanMethylation27 BeadChip was used to obtain methylation profiles for all samples (TIG-3, RIS, and SVts8 cells). An Infinium HumanMethylation450 BeadChip was used to obtain more comprehensive methylation profiles of TIG-3 cells at PDL 36 and PDL 85, and RIS cells. The BeadChip data have been deposited in the GEO (GSE81788 and GSE81797). Scatter plots for β values were drawn using Genome Studio. Hyper- and hypo-methylated CpG sites were classified into six CpG subcategories depending on the location relative to the CpG island (CpG island, N_Shore, S_Shore, N_Shelf, S_Shelf, and the open sea) and into seven gene feature subcategories (− 1500 to − 200 bp upstream of the TSS (TSS1500), − 200 bp to 0 bp upstream of TSS (TSS200), 5’ untranslated region (5’UTR), first exon (1^st^ exon), gene body, 3’ untranslated region (3’UTR), and intergenic region), according to the probe annotation (HumanMethylation450_15017482_v.1.1.csv) provided by Illumina [43].

#### Integrated analysis of DNA methylation and gene expression

Normalized gene expression and DNA methylation array data were integrated based on the genomic locations of the RefSeq genes’ TSS composed in the expression array and the genomic location of the CpG sites placed in the BeadChip array using custom perl scripts. RefSeq information was retrieved from http://hgdownload.cse.ucsc.edu/goldenPath/hg19/database/refGene.txt.gz. We assessed Δβ values for the CpG sites located within 8 kb distance from the closest TSS of the RefSeq genes and the fold-change value of the corresponding gene expression. The gene expression data were obtained from probes annotated by the RefSeq ID for mRNA. When multiple expression probes existed in the same RefSeq ID, the mean of multiple intensities was calculated for the RefSeq ID. The distance from the methylation site to the TSS was calculated with a computer using the location information on the methylation probes and RefSeq (as described above). Promoters registered in RefSeq were classified into two classes, CGI and non-CGI promoters, using the following criteria: > = 50% GC content and observed-to-expected ratio of CpG > = 0.6.

#### Gene ontology (GO) analysis

GO analysis was performed using the Database for Annotation, Visualization and Integrated Discovery (DAVID) v6.7 (http://david.abcc.ncifcrf.gov/) [15–17] with GOTERM_BP_ALL and functional annotation clustering. The genes for GO analysis were extracted according to each cut-off value given in the methylation and integrated analysis section. For GO analysis using only gene expression data, genes exhibiting a more than 3-fold change of expression were used due to the limited gene numbers loaded onto the annotation tool. The top 10 represented GO terms are shown in some tables. Enrichment scores of more than 1.3 gave p-values of less than 0.05.

To conduct GO analysis on gene feature- and CpG site-categories, the genes related to differentially methylated CpG sites on the gene-coding region based on the probe annotation were analyzed with GOTERM_BP_ALL and a functional annotation chart due to the small numbers of extracted genes. The ratios of the number of genes with similar functions were calculated for all gene feature- and CpG site-subcategories.

#### miRNA expression regulated by DNA methylation and its targeted genes

To measure the expression level of miRNAs during replicative senescence, the Human miRNA microarray data were analyzed using GeneSpring (Ver. 12.5). The data were normalized by a 90-percentile shift, and then the ratios to those of the control sample (PDL 44) were obtained. The cut-off for miRNA expression change used in this study was a ±1.5-fold change. The microarray data for miRNA expression have been deposited in the GEO (GSE90942). miRNAs exhibiting methylation changes were obtained from HumanMethylation450 BeadChip data using the “MIR” keyword in the “UCSC_REFGENE_NAME” column provided by Illumina. We searched for miRNAs with hypermethylated promoter regions and down-regulated expression, and vice versa, by comparing methylation changes (Δβ cut-off was ±0.2) with expression changes (fold-change cut-off, ±1.5). Genes targeted by those miRNAs for which expression seemed to be regulated by DNA methylation were picked up by TargetScanHuman Release 7.0. The resulting genes were then further filtered by more than two miRNAs and the gene expression levels based on our microarray data.

## Results

### Similar biological outcomes, but different gene expression patterns were detected in three types of senescent cells

**Fig 1:**
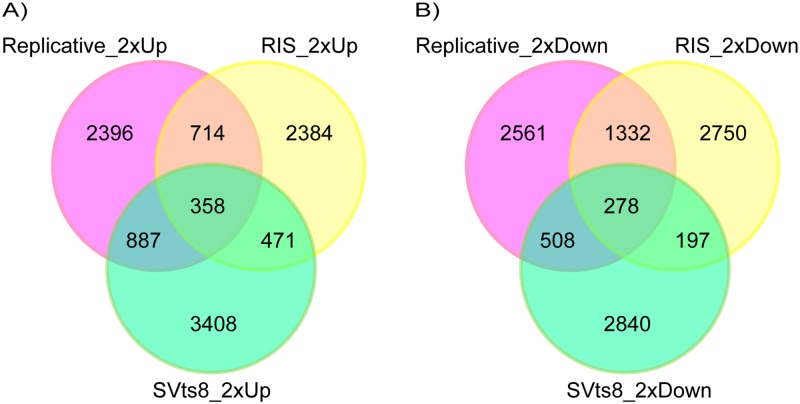
The number of probes indicating up- or down-regulated genes in three types of senescent cells. The number of up-regulated (A) or down-regulated probes (B) are shown in three types of senescent cells. The probes exhibiting a more than 2-fold change were counted. Purple circles, replicatively senescent cells; yellow circles, RIS cells; green circles, senescent SVts8 cells.

**Fig 2:**
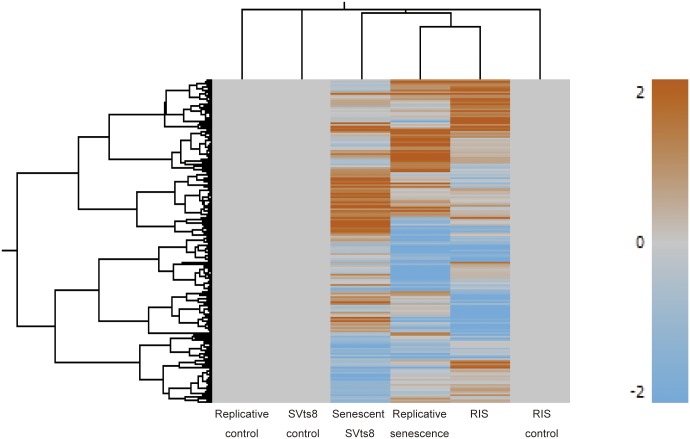
Heatmap of gene expression in three types of senescent cells. Gene expression patterns in three types of senescent cells are shown as a heatmap with sample clustering using uncentered correlation.

We prepared three types of senescent cells, namely, replicatively senescent, RIS and senescent SVts8 cells. The senescence state in SVts8 cells was rapidly induced by the inactivation of the temperature-sensitive mutant of the SV40 large T antigen under non-permissive temperature. Senescence was confirmed by the growth arrest, appearance (a large flat morphology), senescence-associated beta-galactosidase (SA-β-Gal) activity, and protein and mRNA levels of p16 ^INK4A^ and p21^Cip1/Waf1^ (S1 Fig). Gene expression data for senescent cells obtained with SurePrint G3 Human GE microarrays (Agilent) showed that less than 10% of the probes tended to show similar changes in the three types of senescent cells (up-regulated: 358 of 4,355 probes in replicatively senescent cells, 3,927 probes in RIS cells and 5,124 probes in senescent SVts8 cells; down-regulated: 278 of 4,679 probes in replicatively senescent cells, 4,557 probes in RIS cells and 3,823 probes in senescent SVts8 cells), whereas more than 50% of the probes showed changes specific to each type of senescent cell (up-regulated: 2,396 of 4,355 probes in replicatively senescent cells, 2,384 of 3,927 probes in RIS cells, and 3,408 of 5,124 probes in senescent SVts8 cells; down-regulated: 2,561 of 4,679 probes in replicatively senescent cells, 2,750 of 4,557 probes in RIS cells, and 2,840 of 3,823 probes in senescent SVts8 cells) (Fig 1). A heatmap also showed different patterns of gene expression among the three types of senescent cells, even though sample clustering indicated that replicatively senescent cells and RIS cells were closer than senescent SVts8 cells (Fig 2).

According to the GO analysis of more than 3-fold changed genes, the down-regulated genes in the replicatively senescent, RIS and senescent SVts8 cells were mostly related to the “cell cycle” (S1 Table). On the other hand, the up-regulated genes were related to “immune response”, “locomotion”, and “cell migration” in all three types of senescent cells that we examined. Both up-regulated and down-regulated genes included “developmental process” genes. The results suggest that different genes with similar functions contributed to the senescent process, although the biological outcomes were similar among the three types of senescent cells.

### Replicatively senescent cells showed DNA methylation changes

**Fig 3:**
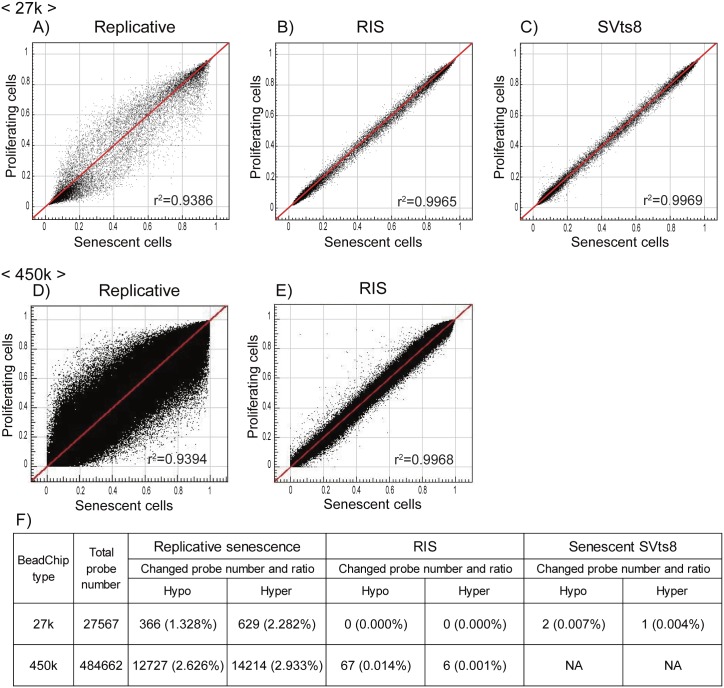
Comparison of DNA methylation profiles in three types of senescent cells. DNA methylation β values of senescent (x-axis) and proliferating control (y-axis) cells in the three types of senescent models are shown in scatter plots. The data were obtained by Infinium HumanMethylation27 (upper panels, A–C) and HumanMethylation450 BeadChip (middle panels, D–E). The correlation coefficient (r^2^) is shown in each plot. The lower table (F) shows the numbers of CpG sites that were hyper- (Δβ > = 0.2) and hypo-methylated (Δβ = < −0.2) upon senescence.

The DNA methylation profiles of senescent and proliferating cells were obtained using the Infinium HumanMethylation27 BeadChip. Among the three types of senescent cells examined, only the replicatively senescent cells (TIG-3 at PDL 85) were notably differentially methylated compared to the proliferating control cells (TIG-3 at PDL 36), whereas prematurely senescent cells (RIS and SVts8) were not (Fig 3, upper panels). Among the 629 and 366 CpG sites that were hyper- and hypo-methylated, respectively, in TIG-3 at PDL 85 (Fig 3, lower table), 565 (89.8%) and 310 (84.7%) sites showed a stepwise increase and decrease, respectively, of DNA methylation along with the progression of PDLs (36, 49, 69, and 85) (Table 1). Genes hosting the differentially methylated CpG sites detected in TIG-3 at PDL 85 were subjected to GO analysis using DAVID. Genes associated with hypomethylated CpG sites were found to be most enriched in the term “immune response” (enrichment score 7.86) and its related terms (S2 Table). On the other hand, genes associated with hypermethylated CpG sites were enriched in a wider variety of terms such as regulation of biological and developmental processes with lower enrichment scores (4.24 or lower).

**Table 1:** Sequential changes of DNA methylation during replicative senescence.

		PDL 49 vs PDL 36	PDL 69 vs PDL 36	PDL 85 vs PDL36
Hypomethylated CpG sites	Total number	64	165	366
	Number of sites with sequential change	–	156 (94.5%)	310(84.7%)
Hypermethylated CpG sites	Total number	76	277	629
	Number of sites with sequential change	–	273 (98.6%)	565 (89.8%)

**Fig 4:**
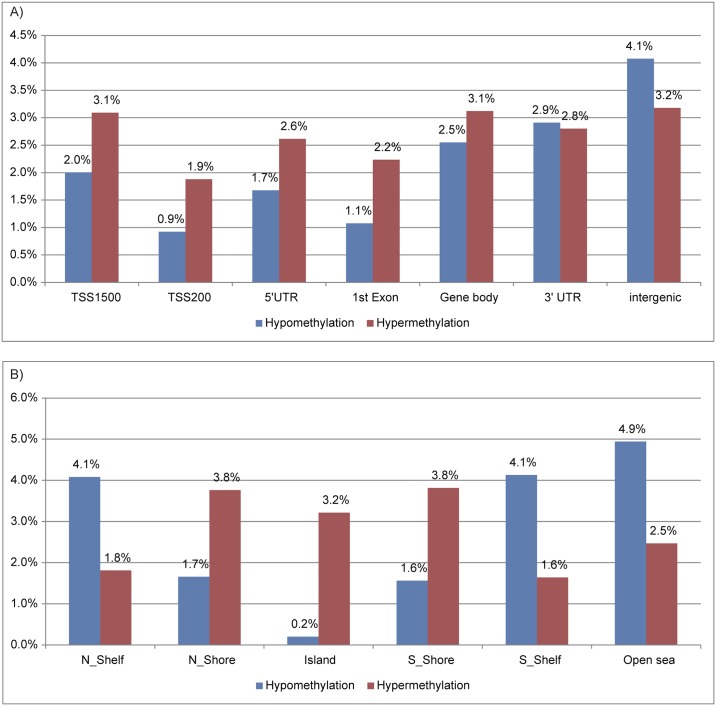
Rates of differentially methylated CpG sites in replicatively senescent TIG-3 cells in seven gene- feature (A) and six CpG- site (B) subcategories. CpG sites showing Δβ > = 0.2 and Δβ = < −0.2 in senescent TIG-3 cells (at PDL 85) compared to those at PDL 36 were regarded as hyper- and hypo-methylated, respectively. Seven gene-feature subcategories: TSS1500, TSS200, 5’UTR, 1^st^ exon, gene body, 3’UTR, and intergenic region. Six CpG subcategories: CpG island, N_Shore, S_Shore, N_Shelf, S_Shelf, and the open sea.

**Fig 5:**
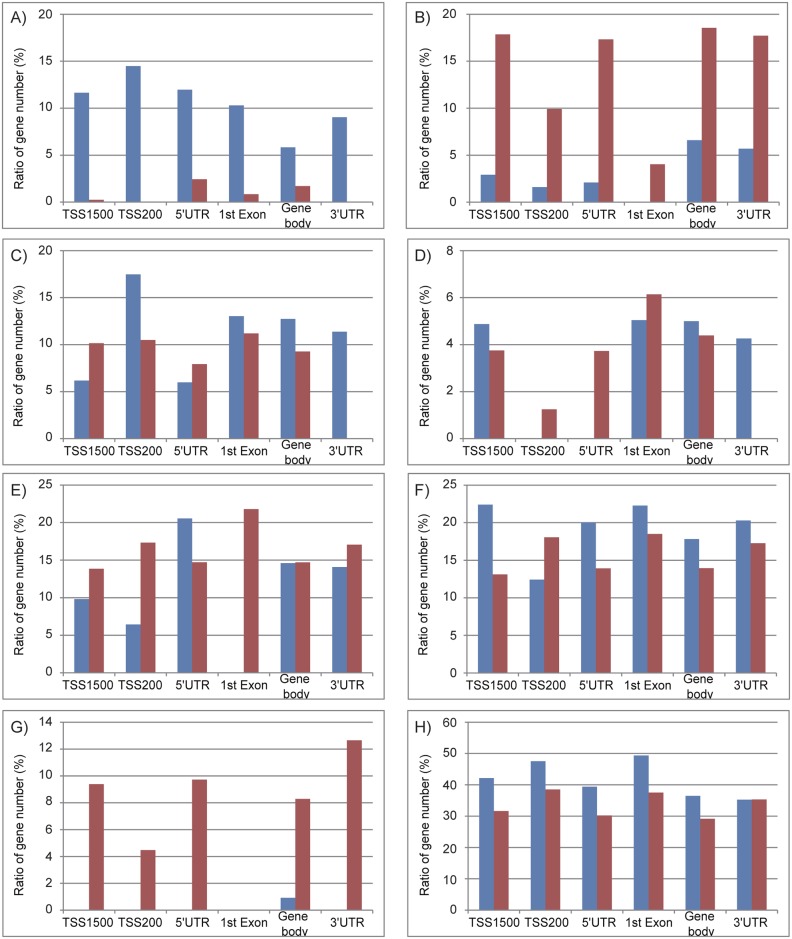
The characterization of genes hosting differentially methylated CpG sites in the gene feature subcategories. GO analyses for gene hosting differentially methylated CpG sites were performed using DAVID (functional annotation chart and GOTERM_BP_ALL). The genes hosting hypermethylated (red) or hypomethylated (blue) CpG sites were classified into six gene feature categories (TSS1500, TSS200, 5’UTR, 1^st^ exon, gene body and 3’UTR). Using the GO terms detected to be enriched (p-value = < 0.05) by DAVID, genes with similar functions were classified into seven groups: immune response (A), metabolic process (B), transport (C), cell adhesion (D), development (E), signal transduction (F), and transcription (G), plus an additional group, others (H). Histograms (A–H) show the ratio of the number of genes classified by GO terms in gene features subcategories (S3 Table shows the number of genes analyzed and classified).

**Fig 6:**
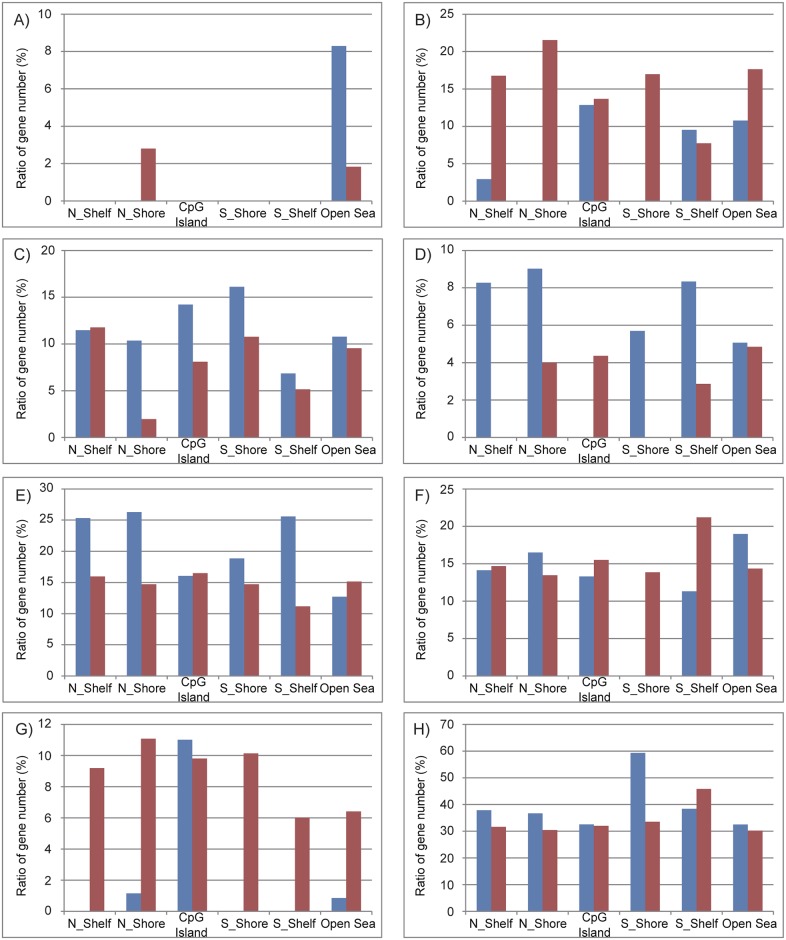
The characterization of genes hosting differentially methylated CpG sites in the CpG site subcategories. The same type of GO analyses shown in Fig 5 was applied to six CpG subcategories (N_Shelf, N_Shore, CpG island, S_Shore, S_Shelf, and the open sea). S3 Table shows the number of genes analyzed and classified.

To more comprehensively examine the alteration of DNA methylation upon senescence, we obtained the DNA methylation profiles of TIG-3 cells at PDL 36 and PDL 85, and RIS cells using the Infinium HumanMethylation450 BeadChip. Again, replicatively senescent TIG-3 cells were differentially methylated compared to the proliferating control cells, whereas RIS cells were not (Fig 3D and 3E). Among 484,662 probes that passed quality control procedures, 14,214 and 12,727 probes were hyper- and hypo-methylated, respectively, in TIG-3 cells at PDL 85 as compared with TIG-3 cells at PDL 36 (Fig 3F). The ratios of differentially methylated probes were determined for each of the seven gene features and six CpG subcategories (Fig 4). The frequency of hypermethylated CpG sites was higher than that of hypomethylated CpG sites in TSS1500 (1.6 fold), TSS200 (2.1 fold), 5’UTR (1.5 fold), 1^st^ exon (2.0 fold) and gene body (1.2 fold) subcategories (Fig 4A). As shown in Fig 4B, the frequency of hypermethylated CpG sites was higher in CGI (16.0 fold), N_Shore (2.2 fold), and S_shore (2.4 fold) subcategories, but was lower in N_Shelf (0.44 fold), S_Shelf (0.39 fold), and the open sea (0.51 fold) subcategories. We applied GO analyses to genes hosting the differentially methylated CpG sites to characterize the features of the genes that were supposed to be regulated in part by DNA methylation in each gene feature- and CpG site-subcategories. A total of 8,114 genes hosting the differentially methylated CpG sites were classified by GO terms into the subcategories. The GO terms obtained from functional annotation charts using DAVID were further categorized into seven groups: “immune response”, “metabolic process”, “transport”, “cell adhesion”, “development”, “signal transduction”, and “transcription”, plus an additional group, others (Figs 5A–5H and 6A–6H and S3 Table). The “immune response”-related genes hosting hypomethylated CpG sites were enriched in all of the gene feature subcategories, whereas a small portion of those hosting hypermethylated sites were located in the TSS1500, 5’UTR, 1^st^ exon and gene body subcategories (Fig 5A). Interestingly, in the region from − 200 bp to 0 bp upstream of TSS (TSS200), all of the classified 63 genes related to “immune response” consisted of the hypomethylated CpG sites. Consistent with the results of GO analyses using HumanMethylation27 data (S2 Table), the genes hosting hypomethylated CpG sites were found to be markedly enriched with the genes involved in “immune response”. In sharp contrast, the “transcription”-related genes hosting hypermethylated CpG sites were enriched in all of the gene feature subcategories except for “1^st^ exon”, whereas no “transcription”-related genes hosting the hypomethylated ones were located in the gene feature subcategories except “gene body” (Fig 5G). The results of the GO analyses conducted on the CpG site subcategories are shown in Fig 6. Genes related to “immune response” were enriched only in the genes hosting hypomethylated CpG sites in the “open sea”, but not those in other subcategories (islands, shores, and shelves) (Fig 6A). In addition, genes related to “transcript” were enriched in the genes hosting hypomethylated CpG sites in the “CpG islands” (Fig 6G). However, genes related to the other six groups of GO terms were enriched in the genes hosting hypomethylated CpGs in several CpG subcategories (Fig 6B–6F and 6H). When we focused on the genes hosting hypermethylated CpG sites, genes related to “immune response” were enriched in “N_shore” rather than in the “open sea”. In addition, genes related to other groups were enriched in the genes hosting hypermethlated CpG sites regardless of the CpG site locations (islands, shores, shelves, and the open sea) (Fig 6B–6G). Thus, a certain portion of “immune response”-related genes might be regulated in part by DNA methylation via CpG sites being outside of the CpG islands.

### Hypomethylation observed in the open sea was frequently associated with the up-regulation of genes related to immune response

Next, we performed an integrated analysis of HumanMethylation450 BeadChip and gene expression profiles to investigate potential functional effects of DNA methylation on gene expression in replicative senescence. Most methylation changes were unlikely to have a significant impact on gene expression (S2 Fig). Out of 212,885 probes located within 8 kb distance from a TSS of RefSeq genes, 1,596 CpG sites were differentially methylated along with the altered expression of the nearest genes to these CpG sites: 1,101 hyper- and 495 hypo-methylated CpG sites for which the nearest genes were down- and up-regulated, respectively, upon senescence (S4 Table). The genes nearest to these 1,596 differentially methylated CpG sites were subjected to GO analysis using DAVID. Those genes with up-regulated expression that were in a hypomethylated state in close proximity (within 8 kb) to their TSS were involved in “immune response” and “cell death” as ranked in the top 10 terms (Table 2). The immune response-related genes included *MHC II*, and the cell death-related genes included *FAS* and *oxidized low density lipoprotein receptor 1 (OLR1)*. In contrast, those genes with down-regulated expression that were in a hypermethylated state in close proximity to their TSS were categorized into several categories such as “development” and “cell cycle”.

**Table 2:** GO analysis of genes showing the relationship between methylation and gene expression level in replicative senescence.

Rank	Hypomethylation & up-regulated gene expression (n = 351)		Hypermethylation & down-regulated gene expression (n = 516)	
	GO term categories	Enrichment Score	GO term categories	Enrichment Score
1	Response to stimulus	3.20	Developmental process	7.08
2	Immune response	3.19	Organ development	5.14
3	Inflammatory response	2.73	Lung alveolus development	3.69
4	Response to hormone stimulus, Response to corticosteroid stimulus	2.50	Embryonic development	3.64
5	Response to nutrient levels, Response to extracellular stimulus	2.30	Cell cycle	3.16
6	Regulation of transport	1.69	Negative regulation of transcription	3.11
7	Developmental process	1.62	Epithelial tube morphogenesis	2.70
8	Cell death	1.37	Mesenchymal cell development	2.47
9	Actin cytoskeleton organization	1.26	Ear development	2.46
10	Negative/positive regulation of kinase activity	1.25	Positive regulation of transcription, DNA-dependent, positive regulation of metabolic process	2.34

* More than 1.3 of enrichment scores gave less than 0.05 p-value.

**Fig 7:**
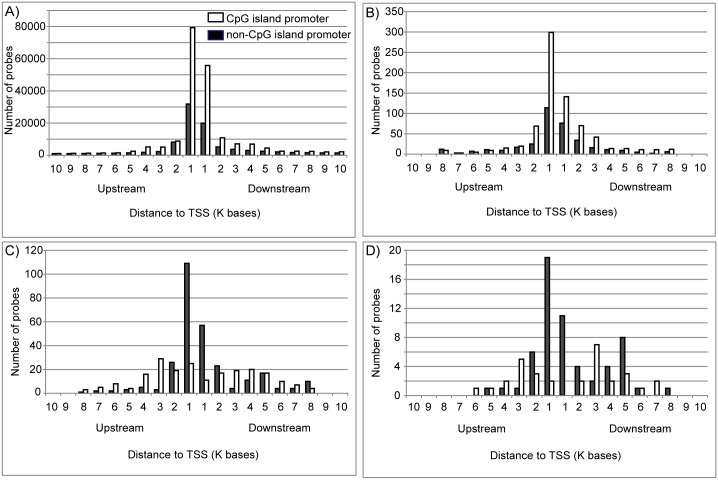
Distribution patterns of the distances between CpG sites and the nearest TSS. Distribution patterns for all 299,261 CpG probes on the HumanMethylation450 BeadChip (A), 1,101 hypermethylated CpG sites for which the nearest gene was down-regulated upon replicative senescence (B), 495 hypomethylated CpG sites for which the nearest gene was up-regulated upon replicative senescence (C), and 90 hypomethylated CpG sites for which the nearest gene was up-regulated upon replicative senescence and related to immune response (D) are shown.

We also assessed the distribution of the distances between each of the 1,596 differentially methylated CpG sites and the nearest TSS. In this analysis, we classified genes into two subcategories based on promoter types, CGI and non-CGI promoters. The number of probes for all CpG sites within CGI and non-CGI promoters on the array were 204,829 and 94,432 respectively (Fig 7A). The number of probes located within 1 kb distance to the TSS was 186,998 (62%). The 1,101 hypermethylated CpG sites for which the nearest gene was down-regulated upon replicative senescence consisted of 744 and 357 CpG sites within CGI and non-CGI promoters, respectively. The hypermethylated CpG sites tended to be located more frequently in CGI promoters than in non-CGI promoters, and located close to the TSS: 630 out of 1,101 (57%) within 1 kb distance (Fig 7B). The distribution patterns were similar between all CpG sites (Fig 7A) and 1,101 hypermethylated CpG sites (Fig 7B). In sharp contrast, 495 hypomethylated CpG sites for which the nearest gene was up-regulated were mainly located in non-CGI promoters (Fig 7C). Within 1 kb distance of the TSS, 202 hypomethylated CpG sites, for which the nearest gene was up-regulated upon replicative senescence, consisted of 36 and 166 CpG sites within CGI and non-CGI promoters, respectively (Fig 7C). This distribution pattern was similar to that of a subset of hypomethylated CpG sites, for which the nearest gene was up-regulated and related to “immune response” (Fig 7D). This result suggests the possibility that DNA methylation on the promoter modulates the expression of a subset of “immune response” genes in replicative senescence.

### DNA methylation affected the expression of miRNAs and the target genes during replicative senescence

A HumanMethylation450 BeadChip includes 3,436 probes related to miRNA. In replicatively senescent TIG-3 cells, 98 CpG sites corresponding to 66 miRNAs were hypomethylated, whereas 102 CpG sites corresponding to 62 miRNAs were hypermethylated (S5 Table). The expression levels of miRNAs were examined using Human miRNA microarray (Release 21.0), where 2,549 human miRNAs were represented. During replicative senescence, 178 miRNAs (fold-change > = ±1.5) showed up- or down-regulated (data not shown). To select miRNAs for which expression seemed to be regulated by DNA methylation, we compared the methylation changes of CpG sites related to miRNA with the miRNA expression changes. Among 18 miRNAs selected, seven miRNAs showed a decrease in expression accompanied with a hypermethylated CpG site in the promoters (S6 Table). This result was consistent with a previous report using IMR 90 cells, where the expression levels of six of the seven miRNAs were down-regulated during replicative senescence [27]. In contrast, no miRNAs showed an increase in expression along with a hypomethylated CpG site in the promoters. We next searched for candidate genes targeted by the seven miRNAs using TargetScan Human Release 7.0, and these genes were further selected by more than two miRNAs and the targeted gene expression levels based on our microarray data. As a result, we identified 27 genes for which expression seemed to be indirectly regulated by DNA methylation on the promoters of targeting miRNAs (Table 3). The genes encoding IL-6 signal transducer (*IL6ST*) and Zinc finger matrin-type 3 (*ZMAT3*) were included in the targeted genes.

**Table 3:** Targeted genes mediated in part by miRNAs, with miRNA expression regulated via methylation in the promoter regions.

Targeted gene symbol	Gene expression (fold-change)	miRNAs of regulating targeted gene		
EIF4EBP2	3.0	hsa-miR-7-5p	hsa-miR-193a-5p	hsa-miR-335-5p
HECW2	4.2	hsa-miR-7-5p	hsa-miR-25-3p	hsa-miR-505-3p
CADM1	8.2	hsa-miR-7-5p	hsa-miR-505-3p	
CNNM2	2.9	hsa-miR-335-5p	hsa-miR-505-3p	
CPEB2	2.6	hsa-miR-7-5p	hsa-miR-505-3p	
CRY2	2.2	hsa-miR-7-5p	hsa-miR-17-5p	
DAAM1	2.5	hsa-miR-130b-3p	hsa-miR-335-5p	
DGKH	2.5	hsa-miR-130b-3p	hsa-miR-505-3p	
DOK6	2.2	hsa-miR-17-5p	hsa-miR-335-5p	
EGR2	2.8	hsa-miR-17-5p	hsa-miR-25-3p	
FAM134C	2.2	hsa-miR-17-5p	hsa-miR-335-5p	
GRIN2A	2.0	hsa-miR-7-5p	hsa-miR-130b-3p	
HPCAL4	7.6	hsa-miR-7-5p	hsa-miR-335-5p	
IL6ST	2.2	hsa-miR-130b-3p	hsa-miR-505-3p	
KLHL28	2.4	hsa-miR-7-5p	hsa-miR-335-5p	
MEF2D	2.7	hsa-miR-335-5p	hsa-miR-505-3p	
MYO1D	5.9	hsa-miR-193a-5p	hsa-miR-335-5p	
NR4A3	2.1	hsa-miR-7-5p	hsa-miR-335-5p	
OXR1	2.9	hsa-miR-7-5p	hsa-miR-17-5p	
PGM2L1	4.8	hsa-miR-17-5p	hsa-miR-130b-3p	
PIP4K2C	2.1	hsa-miR-25-3p	hsa-miR-505-3p	
PPARGC1B	2.6	hsa-miR-7-5p	hsa-miR-505-3p	
RAB11FIP5	2.8	hsa-miR-7-5p	hsa-miR-17-5p	
RNF141	2.4	hsa-miR-7-5p	hsa-miR-335-5p	
SEMA6D	5.9	hsa-miR-7-5p	hsa-miR-193a-5p	
ZDHHC8	5.2	hsa-miR-17-5p	hsa-miR-335-5p	
ZMAT3	3.4	hsa-miR-7-5p	hsa-miR-130b-3p	

## Discussion

In this study, we examined DNA methylation levels in three types of senescent cells and found that only replicatively senescent cells were differentially methylated, whereas prematurely senescent ones (RIS and SVts8) were not (Fig 3 and Table 1). These results were in good agreement with previous studies showing DNA methylation changes in aged tissues and cells [16, 21, 24, 44]. There are several reasons why the DNA methylation profile is strongly modified during replicative senescence and not during premature senescence. Firstly, errors may accumulate due to repeated cell division. Laird *et al*. evaluated the fidelity of transmission of the DNA methylation state in the CpG island of the *FMR1* gene in normal human lymphocytes using hairpin-bisulfite PCR [45]. Although the high fidelity of inheritance of the methylated state of cytosine was estimated, the results clearly showed that errors in maintaining DNA methylation occurred to some extent in every DNA replication. When culturing TIG-3 cells from PDL 36 to PDL 85, the cells would be divided approximately 2^50 times. In contrast, as RIS and senescent SVts8 cells were rapidly induced to a senescent state, prematurely senescent cells would not have enough rounds of cell division to accumulate any errors. Secondly, reduced activity of DNA methyltransferase 1 (DNMT1), which primarily maintains DNA methylation patterns during replication, may increase the incidence of errors. The expression profiles in this study exhibited a marked decrease in *DNMT1* in replicatively senescent cells (0.23-fold reduction, data not shown), whereas the expression levels of *DNMT1* in RIS (0.70-fold reduction, data not shown) and senescent SVts8 (0.80-fold reduction, data not shown) were slightly reduced. This is consistent with the results reported by Kaneda *et al*., who reported that DNA methylation was not altered in RIS using methylated DNA immunoprecipitation (MeDIP) sequencing and bisulfite sequencing, and the *Dnmt1* expression level was not altered in RIS, or during 3 passages (passage 2 to 5) using mouse embryonic fibroblasts [16]. Thirdly, a marked decrease in Ten-eleven translocation 1 (TET1) expression could alter DNA methylation patterns. Recent studies have suggested active DNA demethylation is mediated by TET, the enzyme that converts 5-methylcytosine (5mC) to 5-hydroxymethylcytosine (5hmC), 5-formylcytosine (5fc), and 5-carboxylcytosine (5caC) [46–49]. There are three TET proteins: TET1, TET2 and TET3. The expression levels of *TET1* and *TET3* genes, but not *TET2*, reportedly decrease along with aging, and *TET3* expression is important for decreasing genomic 5hmC during aging in human T cells [50]. In replicatively senescent TIG-3 cells, the expression levels of the *TET1* gene were drastically decreased, although we have no expression data for the TET3 gene due to a lack of TET3 probes in the expression array. A recent study showed that *Tet1*/*Tet2* double-knockout mouse embryonic fibroblasts (MEFs) had defects in maintaining hypomethylation and resulted in hypermethylation of DNA methylation canyons where developmental genes are associated [51]. Taken togather, methylation changes during senescence would be required for many rounds of cell division and the decreased activities of DNA methyltransferase and methylcytsine deoxygenase, such as DNMT1 and TET1, might produce the altered methylation patterns within long-term culture.

We found that hypomethylation observed in the open sea was frequently associated with the up-regulation of genes related to immune response. When the genes hosting hypomethylated CpG sites were classified into six CpG subcategories using annotated GO terms, genes related to “immune response” were enriched only in the genes hosting hypomethylated CpG sites in the “open sea” (Fig 6A). Consistent with the results, the promoter types of genes categorized as “immune response” were mainly non-CGI promoters (Fig 7D). Nevertheless, the probes in the HumanMethylation450 BeadChip covered much more CpG island promoter genes than non-CpG island promoter genes (Fig 7A). In contrast, genes categorized into other groups, namely “metabolic process”, “transport”, “cell adhesion”, “development”, “signal transduction”, and “transcription”, exhibited hypermethylation rather than hypomethylation in the CpG sites close to the TSS (S3 Table). Furthermore, integrated analyses showed that “immune response” was ranked in GO terms of genes up-regulated and concomitantly hypomethylated in close proximity to their TSS (Table 2). These results suggest that hypomethylation in the open sea may increase the expression of the “immune response”-related genes during replicative senescence. For example, *MHC II* has non-CGI promoters exhibiting hypomethylated CpG sites and its expression was up-regulated during replicative senescence. Several studies also indicated the effects of DNA methylation in the promoter regions on the expression of inflammatory genes [52–54].

Currently we cannot explain the mechanism by which hypomethylation in the non-CpG promoter increases the expression of a certain portion of the “immune response”-related genes during replicative senescence. We speculate that TET2 may play a role in this regulation. As mentioned above, TET proteins contribute to active DNA demethylation. In our data, the expression levels of one of the *TET2* probes showed a 2-fold increase in replicatively senescent cells and senescent SVts8 cells, whereas no increase in *TET2* expression was detected in the RIS cells. Unlike TET1 and TET3, TET2 does not have a CXXC domain, which is required for binding to the CpG site [55, 56]. These results suggest that collaboration between TET2 and its associated factor(s) may be required for DNA binding and demethylation. In fact, TET2 was reported to need a cofactor for binding to DNA [49]. During differentiation of helper T (Th) cells, TET2 induced DNA demethylation at the loci of key cytokine genes in a lineage-specific transcription factor-dependent manner and promoted signature cytokine expression in Th1 and Th17 cells [57]. Depending on the cofactor, TET2 may bind to non-CGI promoters in senescence-associated genes, and increase gene expression. Further studies are needed to investigate the effects of TET2 on hypomethylation at the specific CpG sites and its interacting factor(s) during senescence.

Recently, senescence-associated miRNAs (SA-miRNAs) have been reported to regulate genes associated with senescence [27–31]. We examined the effects of DNA methylation in the promoter regions of miRNAs on miRNA expression using human miRNA expression microarray and further selected miRNAs that likely affected the predicted gene expression. As a result, we identified seven miRNAs and 27 targeted genes (Table 3). These genes included eukaryotic translation initiation factor 4E binding protein 2 (*EIF4EBP2*), which inhibits translation initiation [58], and cryptochrome circadian clock 2 (*CRY2*), which is a key component in regulating circadian rhythm [59]. Among the 27 genes, the IL-6 signal transducer (*IL6ST*) and zinc finger matrin-type 3 (*ZMAT3*) are involved in immune response. *IL6ST*, which is supposed to be up-regulated by decreased levels of has-miR-130b-3p and has-miR-505-3p, encodes glycoprotein 130 (gp130) [60]. The protein gp130 is a signal transducer shared by many cytokines including IL-6, one of the SASP factors [61], IL-11, IL-27, and oncostatin-M [62–64]. In addition, *ZMAT3*, a predicted gene regulated by has-miR-7-5p and has-miR-130b-3p, encodes a double-stranded-RNA-binding zinc finger protein Wig-1 (for wild-type p53-induced gene 1). Wig-1, a transcriptional target of p53, stabilizes p53 by binding to the 3’ UTR of p53 mRNA and protecting it from deadenylation [65]. A high level of p53 triggers cell cycle arrest, senescence and apoptosis, and efficiently inhibits tumor development [7, 66, 67]. In addition to the p53 transcription factor, miRNAs with expression regulated by DNA methylation via their promoter regions may also contribute to *ZMAT3* expression. MiR-34 is one of the SA-miRNAs and is up-regulated by p53 [27, 28, 32, 33, 68, 69]. Although increased levels of miRNA-34 were detected upon replicative senescence, the methylation changes (Δβ cut-off was ±0.2) in the promoter regions of miRNA-34 were not included in our data (data not shown).

In this study, we investigated the possibility of regulation by DNA methylation during senescence. We found that hypomethylation in the open sea may contribute to the up-regulation of genes related to immune response. Several miRNAs targeting genes associated with a senescent state seem to regulate expression by DNA methylation in the promoter regions. However, we have to consider the possibility that DNA methylation results from alteration of gene expression. To investigate this possibility, we need to collect more data and explore the mechanism of methylation and demethylation change. Moreover, in order to reveal the whole mechanism of DNA methylation, we also need to focus on the methylation profile in hypomethylation and outside of the TSS and CpG islands.

## Conclusion

Three types of senescent TIG-3 cells showed similar biological outcomes, but the regulatory mechanisms were different. Replicatively senescent cells showed sequential DNA methylation changes, but prematurely senescent RIS and SVts8 cells did not. In replicative senescence, hypomethylation with up-regulated gene expression often occurred in the open sea. Moreover, hypomethylation was observed in non-CGI promoters of genes related to the immune response. These results suggested that hypomethylation in the open sea regulates the expression of a certain portion of immune-related genes in replicative senescence. In addition, several miRNAs that seemed to have expression levels regulated in part by DNA methylation may also contribute to the expression of senescence-associated genes.

## Supporting information

S1 Fig Confirmation of senescence. Senescent cells were subjected to senescence-associated beta-galactosidase (SA-β-Gal) staining, qRT-PCR and immunoblotting. SA-β-Gal staining of the control A), C), E), replicatively senescent B), RIS D), and senescent SVts8 cells F). The percentages of SA-β-Gal-positive cells are shown at the bottom of each picture. Bar, 200 μm. Objective, ×10. G) The expression levels of p16^INK4A^ and p21^Cip1/Waf1^ obtained with SurePrint G3 Human GE microarrays and qRT-PCR. H) Representative western blotting of p16^INK4A^ and p21^Cip1/Waf1^, Ras, and loading control (actin). Images of p16^INK4A^ are shown separately shown due to different exposure time.
(TIF)

S2 Fig Integrated analysis of methylation and gene expression in replicative senescence. Each plot represents the values obtained from a single gene using integrated analyses. DNA methylation β values and gene expression levels are plotted along the abscissa and the ordinate, respectively. Red lines show the cut-off border. For methylation, Δβ for hypermethylation is > = 0.2, Δβ for hypomethylation is = < −0.2. For gene expression, the cut-off for increased/decreased expression was a ±2-fold change.
(TIF)

S1 Table GO terms for up- or down-regulated genes in three types of senescent cells. (XLSX)

S2 Table GO terms for hypo- or hyper-methylated genes in replicatively senescent cells. (XLSX)

S3 Table The number of genes hosting differentially methylated CpG sites in the gene feature- and the CpG site- subcategories. (XLSX)

S4 Table Integrated analysis of gene expression and methylation changes in replicatively senescent cells. (ZIP)

S5 Table Hypo- or hyper-methylated miRNAs in the promoter regions of replicatively senescent cells. (XLSX)

S6 Table miRNA expression regulated by DNA methylation in the promoter region. (XLSX)

## References

[1] Hayflick L. The limited in vitro lifetime of human diploid cell strains. Exp Cell Res. 1965;37:614–636. 14315085 10.1016/0014-4827(65)90211-9

[2] Kuilman T, Michaloglou C, Mooi WJ, Peeper DS. The essence of senescence. Genes Dev. 2010;24(22):2463–2479. 10.1101/gad.1971610 21078816 PMC2975923

[3] Harley CB, Futcher AB, Greider CW. Telomeres shorten during ageing of human fibroblasts. Nature. 1990;345(6274):458–460. 10.1038/345458a0 2342578

[4] Serrano M, Lin AW, McCurrach ME, Beach D, Lowe SW. Oncogenic ras provokes premature cell senescence associated with accumulation of p53 and p16INK4a. Cell. 1997;88(5):593–602. 9054499 10.1016/s0092-8674(00)81902-9

[5] Takahashi A, Ohtani N, Yamakoshi K, Iida S, Tahara H, Nakayama K, et al Mitogenic signalling and the p16INK4a-Rb pathway cooperate to enforce irreversible cellular senescence. Nat Cell Biol. 2006;8(11):1291–1297. 10.1038/ncb1491 17028578

[6] Collado M, Blasco MA, Serrano M. Cellular senescence in cancer and aging. Cell. 2007;130(2):223–233. 10.1016/j.cell.2007.07.003 17662938

[7] Campisi J. Cellular senescence as a tumor-suppressor mechanism. Trends Cell Biol. 2001;11(11):S27–31. 11684439 10.1016/s0962-8924(01)02151-1

[8] Campisi J. Aging, cellular senescence, and cancer. Annu Rev Physiol. 2013;75:685–705. 10.1146/annurev-physiol-030212-183653 23140366 PMC4166529

[9] Coppe JP, Patil CK, Rodier F, Sun Y, Munoz DP, Goldstein J, et al Senescence-associated secretory phenotypes reveal cell-nonautonomous functions of oncogenic RAS and the p53 tumor suppressor. PLoS Biol. 2008;6(12):2853–2868. 10.1371/journal.pbio.0060301 19053174 PMC2592359

[10] Wu Z, Nakanishi H. Lessons from Microglia Aging for the Link between Inflammatory Bone Disorders and Alzheimer's Disease. J Immunol Res. 2015;2015:1–9. 10.1155/2015/471342 PMC4452354 26078980

[11] Narita M, Nunez S, Heard E, Narita M, Lin AW, Hearn SA, et al Rb-mediated heterochromatin formation and silencing of E2F target genes during cellular senescence. Cell. 2003;113(6):703–716. 12809602 10.1016/s0092-8674(03)00401-x

[12] Narita M, Narita M, Krizhanovsky V, Nunez S, Chicas A, Hearn SA, et al A novel role for high-mobility group a proteins in cellular senescence and heterochromatin formation. Cell. 2006;126(3):503–514. 10.1016/j.cell.2006.05.052 16901784

[13] Zhang W, Hu D, Ji W, Yang L, Yang J, Yuan J, et al Histone modifications contribute to cellular replicative and hydrogen peroxide-induced premature senescence in human embryonic lung fibroblasts. Free Radic Res. 2014;48(5):550–559. 10.3109/10715762.2014.893580 24528089

[14] Shah PP, Donahue G, Otte GL, Capell BC, Nelson DM, Cao K, et al Lamin B1 depletion in senescent cells triggers large-scale changes in gene expression and the chromatin landscape. Genes Dev. 2013;27(16):1787–1799. 10.1101/gad.223834.113 23934658 PMC3759695

[15] Bracken AP, Kleine-Kohlbrecher D, Dietrich N, Pasini D, Gargiulo G, Beekman C, et al The Polycomb group proteins bind throughout the INK4A-ARF locus and are disassociated in senescent cells. Genes Dev. 2007;21(5):525–530. 10.1101/gad.415507 17344414 PMC1820894

[16] Kaneda A, Fujita T, Anai M, Yamamoto S, Nagae G, Morikawa M, et al Activation of Bmp2-Smad1 signal and its regulation by coordinated alteration of H3K27 trimethylation in Ras-induced senescence. PLoS Genet. 2011;7(11):e1002359 10.1371/journal.pgen.1002359 22072987 PMC3207904

[17] Takahashi A, Imai Y, Yamakoshi K, Kuninaka S, Ohtani N, Yoshimoto S, et al DNA damage signaling triggers degradation of histone methyltransferases through APC/C(Cdh1) in senescent cells. Mol Cell. 2012;45(1):123–131. 10.1016/j.molcel.2011.10.018 22178396

[18] Zhang Y, Elgizouli M, Schottker B, Holleczek B, Nieters A, Brenner H. Smoking-associated DNA methylation markers predict lung cancer incidence. Clin Epigenetics. 2016;8(127):1–12. 27924164 10.1186/s13148-016-0292-4 PMC5123284

[19] Asada K, Kotake Y, Asada R, Saunders D, Broyles RH, Towner RA, et al LINE-1 hypomethylation in a choline-deficiency-induced liver cancer in rats: dependence on feeding period. J Biomed Biotechnol. 2006;2006(1):1–6. 10.1155/JBB/2006/17142 PMC1479888 16877811

[20] Varley KE, Gertz J, Bowling KM, Parker SL, Reddy TE, Pauli-Behn F, et al Dynamic DNA methylation across diverse human cell lines and tissues. Genome Res. 2013;23(3):555–567. 10.1101/gr.147942.112 23325432 PMC3589544

[21] Horvath S, Zhang Y, Langfelder P, Kahn RS, Boks MP, van Eijk K, et al Aging effects on DNA methylation modules in human brain and blood tissue. Genome Biol. 2012;13(10):1–18. 10.1186/gb-2012-13-10-r97 PMC4053733 23034122

[22] Koch CM, Wagner W. Epigenetic biomarker to determine replicative senescence of cultured cells. Methods Mol Biol. 2013;1048:309–321. 10.1007/978-1-62703-556-9_20 23929112

[23] Koch CM, Suschek CV, Lin Q, Bork S, Goergens M, Joussen S, et al Specific age-associated DNA methylation changes in human dermal fibroblasts. PLoS One. 2011;6(2):e16679 10.1371/journal.pone.0016679 21347436 PMC3035656

[24] Christensen BC, Houseman EA, Marsit CJ, Zheng S, Wrensch MR, Wiemels JL, et al Aging and environmental exposures alter tissue-specific DNA methylation dependent upon CpG island context. PLoS Genet. 2009;5(8):e1000602 10.1371/journal.pgen.1000602 19680444 PMC2718614

[25] Fraga MF, Ballestar E, Paz MF, Ropero S, Setien F, Ballestar ML, et al Epigenetic differences arise during the lifetime of monozygotic twins. Proc Natl Acad Sci U S A. 2005;102(30):10604–10609. 10.1073/pnas.0500398102 16009939 PMC1174919

[26] Jjingo D, Conley AB, Yi SV, Lunyak VV, Jordan IK. On the presence and role of human gene-body DNA methylation. Oncotarget. 2012;3(4):462–474. 10.18632/oncotarget.497 22577155 PMC3380580

[27] Dhahbi JM, Atamna H, Boffelli D, Magis W, Spindler SR, Martin DI. Deep sequencing reveals novel microRNAs and regulation of microRNA expression during cell senescence. PLoS One. 2011;6(5):e20509 10.1371/journal.pone.0020509 21637828 PMC3102725

[28] Lafferty-Whyte K, Cairney CJ, Jamieson NB, Oien KA, Keith WN. Pathway analysis of senescence-associated miRNA targets reveals common processes to different senescence induction mechanisms. Biochim Biophys Acta. 2009;1792(4):341–352. 10.1016/j.bbadis.2009.02.003 19419692

[29] Taguchi YH. Inference of Target Gene Regulation via miRNAs during Cell Senescence by Using the MiRaGE Server. Aging Dis. 2012;3(4):301–306. 23185711 PMC3501365

[30] Overhoff MG, Garbe JC, Koh J, Stampfer MR, Beach DH, Bishop CL. Cellular senescence mediated by p16INK4A-coupled miRNA pathways. Nucleic Acids Res. 2014;42(3):1606–1618. 10.1093/nar/gkt1096 24217920 PMC3919591

[31] Li CW, Wang WH, Chen BS. Investigating the specific core genetic-and-epigenetic networks of cellular mechanisms involved in human aging in peripheral blood mononuclear cells. Oncotarget. 2016;7(8):8556–8579. 10.18632/oncotarget.7388 26895224 PMC4890987

[32] Tazawa H, Tsuchiya N, Izumiya M, Nakagama H. Tumor-suppressive miR-34a induces senescence-like growth arrest through modulation of the E2F pathway in human colon cancer cells. Proc Natl Acad Sci U S A. 2007;104(39):15472–15477. 10.1073/pnas.0707351104 17875987 PMC2000550

[33] He X, He L, Hannon GJ. The guardian's little helper: microRNAs in the p53 tumor suppressor network. Cancer Res. 2007;67(23):11099–11101. 10.1158/0008-5472.CAN-07-2672 18056431

[34] Christoffersen NR, Shalgi R, Frankel LB, Leucci E, Lees M, Klausen M, et al p53-independent upregulation of miR-34a during oncogene-induced senescence represses MYC. Cell Death Differ. 2010;17(2):236–245. 10.1038/cdd.2009.109 19696787

[35] Ayala-Ortega E, Arzate-Mejia R, Perez-Molina R, Gonzalez-Buendia E, Meier K, Guerrero G, et al Epigenetic silencing of miR-181c by DNA methylation in glioblastoma cell lines. BMC Cancer. 2016;16(226):1–12. 10.1186/s12885-016-2273-6 PMC4794844 26983574

[36] Asuthkar S, Velpula KK, Chetty C, Gorantla B, Rao JS. Epigenetic regulation of miRNA-211 by MMP-9 governs glioma cell apoptosis, chemosensitivity and radiosensitivity. Oncotarget. 2012;3(11):1439–1454. 10.18632/oncotarget.683 23183822 PMC3717804

[37] Yin H, Song P, Su R, Yang G, Dong L, Luo M, et al DNA Methylation mediated down-regulating of MicroRNA-33b and its role in gastric cancer. Sci Rep. 2016;6(18824):1–12. 26729612 10.1038/srep18824 PMC4700416

[38] Maehara K, Takahashi K, Saitoh S. CENP-A reduction induces a p53-dependent cellular senescence response to protect cells from executing defective mitoses. Mol Cell Biol. 2010;30(9):2090–2104. 10.1128/MCB.01318-09 20160010 PMC2863584

[39] Chen C, Okayama H. High-efficiency transformation of mammalian cells by plasmid DNA. Mol Cell Biol. 1987;7(8):2745–2752. 3670292 10.1128/mcb.7.8.2745-2752.1987 PMC367891

[40] Tsuyama N, Miura M, Kitahira M, Ishibashi S, Ide T. SV40 T-antigen is required for maintenance of immortal growth in SV40-transformed human fibroblasts. Cell Struct Funct. 1991;16(1):55–62. 1851674 10.1247/csf.16.55

[41] Bibikova M, Le J, Barnes B, Saedinia-Melnyk S, Zhou L, Shen R, et al Genome-wide DNA methylation profiling using Infinium(R) assay. Epigenomics. 2009;1(1):177–200. 10.2217/epi.09.14 22122642

[42] Bibikova M, Barnes B, Tsan C, Ho V, Klotzle B, Le JM, et al High density DNA methylation array with single CpG site resolution. Genomics. 2011;98(4):288–295. 10.1016/j.ygeno.2011.07.007 21839163

[43] Price ME, Cotton AM, Lam LL, Farre P, Emberly E, Brown CJ, et al Additional annotation enhances potential for biologically-relevant analysis of the Illumina Infinium HumanMethylation450 BeadChip array. Epigenetics Chromatin. 2013;6(4):1–15. 23452981 10.1186/1756-8935-6-4 PMC3740789

[44] Hannum G, Guinney J, Zhao L, Zhang L, Hughes G, Sadda S, et al Genome-wide methylation profiles reveal quantitative views of human aging rates. Mol Cell. 2013;49(2):359–367. 10.1016/j.molcel.2012.10.016 23177740 PMC3780611

[45] Laird CD, Pleasant ND, Clark AD, Sneeden JL, Hassan KM, Manley NC, et al Hairpin-bisulfite PCR: assessing epigenetic methylation patterns on complementary strands of individual DNA molecules. Proc Natl Acad Sci U S A. 2004;101(1):204–209. 10.1073/pnas.2536758100 14673087 PMC314163

[46] Chen ZX, Riggs AD. DNA methylation and demethylation in mammals. J Biol Chem. 2011;286(21):18347–18353. 10.1074/jbc.R110.205286 21454628 PMC3099650

[47] Wu SC, Zhang Y. Active DNA demethylation: many roads lead to Rome. Nat Rev Mol Cell Biol. 2010;11(9):607–620. 10.1038/nrm2950 20683471 PMC3711520

[48] Weber AR, Krawczyk C, Robertson AB, Kusnierczyk A, Vagbo CB, Schuermann D, et al Biochemical reconstitution of TET1-TDG-BER-dependent active DNA demethylation reveals a highly coordinated mechanism. Nat Commun. 2016;7(10806):1–13. 10.1038/ncomms10806 PMC4778062 26932196

[49] Pastor WA, Aravind L, Rao A. TETonic shift: biological roles of TET proteins in DNA demethylation and transcription. Nat Rev Mol Cell Biol. 2013;14(6):341–356. 10.1038/nrm3589 23698584 PMC3804139

[50] Truong TP, Sakata-Yanagimoto M, Yamada M, Nagae G, Enami T, Nakamoto-Matsubara R, et al Age-Dependent Decrease of DNA Hydroxymethylation in Human T Cells. J Clin Exp Hematop. 2015;55(1):1–6. 10.3960/jslrt.55.1 26105999

[51] Wiehle L, Raddatz G, Musch T, Dawlaty MM, Jaenisch R, Lyko F, et al Tet1 and Tet2 Protect DNA Methylation Canyons against Hypermethylation. Mol Cell Biol. 2015;36(3):452–461. 10.1128/MCB.00587-15 26598602 PMC4719427

[52] Oliveira NF, Damm GR, Andia DC, Salmon C, Nociti FH Jr., Line SR, et al DNA methylation status of the IL8 gene promoter in oral cells of smokers and non-smokers with chronic periodontitis. J Clin Periodontol. 2009;36(9):719–725. 10.1111/j.1600-051X.2009.01446.x 19659670

[53] Rusiecki JA, Byrne C, Galdzicki Z, Srikantan V, Chen L, Poulin M, et al PTSD and DNA Methylation in Select Immune Function Gene Promoter Regions: A Repeated Measures Case-Control Study of U.S. Military Service Members. Front Psychiatry. 2013;4:1–12. 23805108 10.3389/fpsyt.2013.00056 PMC3690381

[54] Ushijima T. Epigenetic field for cancerization. J Biochem Mol Biol. 2007;40(2):142–150. 17394762 10.5483/bmbrep.2007.40.2.142

[55] Lee JH, Voo KS, Skalnik DG. Identification and characterization of the DNA binding domain of CpG-binding protein. J Biol Chem. 2001;276(48):44669–44676. 10.1074/jbc.M107179200 11572867

[56] Williams K, Christensen J, Helin K. DNA methylation: TET proteins-guardians of CpG islands? EMBO Rep. 2012;13(1):28–35. 10.1038/embor.2011.233 PMC3246258 22157888

[57] Ichiyama K, Chen T, Wang X, Yan X, Kim BS, Tanaka S, et al The methylcytosine dioxygenase Tet2 promotes DNA demethylation and activation of cytokine gene expression in T cells. Immunity. 2015;42(4):613–626. 10.1016/j.immuni.2015.03.005 25862091 PMC4956728

[58] Banko JL, Poulin F, Hou L, DeMaria CT, Sonenberg N, Klann E. The translation repressor 4E-BP2 is critical for eIF4F complex formation, synaptic plasticity, and memory in the hippocampus. J Neurosci. 2005;25(42):9581–9590. 10.1523/JNEUROSCI.2423-05.2005 16237163 PMC6725736

[59] van der Horst GT, Muijtjens M, Kobayashi K, Takano R, Kanno S, Takao M, et al Mammalian Cry1 and Cry2 are essential for maintenance of circadian rhythms. Nature. 1999;398(6728):627–630. 10.1038/19323 10217146

[60] Rose-John S, Heinrich PC. Soluble receptors for cytokines and growth factors: generation and biological function. Biochem J. 1994;300:281–290. 8002928 10.1042/bj3000281 PMC1138158

[61] Freund A, Orjalo AV, Desprez PY, Campisi J. Inflammatory networks during cellular senescence: causes and consequences. Trends Mol Med. 2010;16(5):238–246. 10.1016/j.molmed.2010.03.003 20444648 PMC2879478

[62] Baumann H, Schendel P. Interleukin-11 regulates the hepatic expression of the same plasma protein genes as interleukin-6. J Biol Chem. 1991;266(30):20424–20427. 1718962

[63] Heinrich PC, Behrmann I, Haan S, Hermanns HM, Muller-Newen G, Schaper F. Principles of interleukin (IL)-6-type cytokine signalling and its regulation. Biochem J. 2003;374(Pt 1):1–20. 10.1042/BJ20030407 12773095 PMC1223585

[64] Pflanz S, Hibbert L, Mattson J, Rosales R, Vaisberg E, Bazan JF, et al WSX-1 and glycoprotein 130 constitute a signal-transducing receptor for IL-27. J Immunol. 2004;172(4):2225–2231. 14764690 10.4049/jimmunol.172.4.2225

[65] Vilborg A, Glahder JA, Wilhelm MT, Bersani C, Corcoran M, Mahmoudi S, et al The p53 target Wig-1 regulates p53 mRNA stability through an AU-rich element. Proc Natl Acad Sci U S A. 2009;106(37):15756–15761. 10.1073/pnas.0900862106 19805223 PMC2773521

[66] Beausejour CM, Krtolica A, Galimi F, Narita M, Lowe SW, Yaswen P, et al Reversal of human cellular senescence: roles of the p53 and p16 pathways. EMBO J. 2003;22(16):4212–4222. 10.1093/emboj/cdg417 12912919 PMC175806

[67] Vousden KH. Outcomes of p53 activation—spoilt for choice. J Cell Sci. 2006;119(Pt 24):5015–5020. 10.1242/jcs.03293 17158908

[68] Disayabutr S, Kim EK, Cha SI, Green G, Naikawadi RP, Jones KD, et al miR-34 miRNAs Regulate Cellular Senescence in Type II Alveolar Epithelial Cells of Patients with Idiopathic Pulmonary Fibrosis. PLoS One. 2016;11(6):e0158367 10.1371/journal.pone.0158367 27362652 PMC4928999

[69] Harries LW. MicroRNAs as Mediators of the Ageing Process. Genes (Basel). 2014;5(3):656–670. 25140888 10.3390/genes5030656 PMC4198923

