# Supplementary figures and images for: Potential roles of DNA methylation in the initiation and establishment of replicative senescence revealed by array-based methylome and transcriptome analyses
Source: PLoS One. 2017 Feb 3;12(2):e0171431. doi: 10.1371/journal.pone.0171431 (PMC5291461; doi:10.1371/journal.pone.0171431)

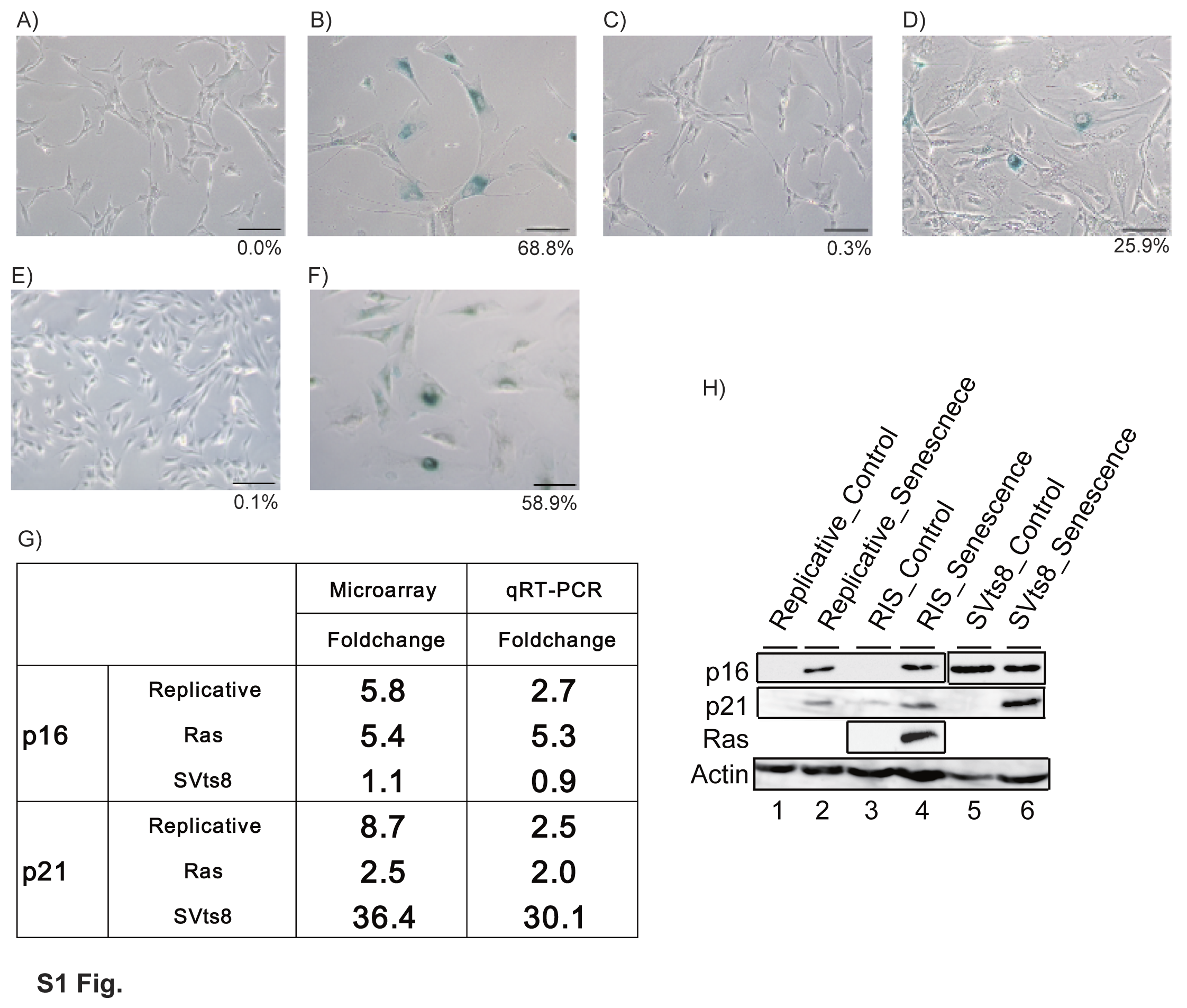

Supplement: S1 Fig — Senescent cells were subjected to senescence-associated beta-galactosidase (SA-β-Gal) staining, qRT-PCR and immunoblotting. SA-β-Gal staining of the control A), C), E), replicatively senescent B), RIS D), and senescent SVts8 cells F). The percentages of SA-β-Gal-positive cells are shown at the bottom of each picture. Bar, 200 μm. Objective, ×10. G) The expression levels of p16INK4A and p21Cip1/Waf1 obtained with SurePrint G3 Human GE microarrays and qRT-PCR. H) Representative western blotting of p16INK4A and p21Cip1/Waf1, Ras, and loading control (actin). Images of p16INK4A are shown separately shown due to different exposure time. (TIF) [file pone.0171431.s001.tif]

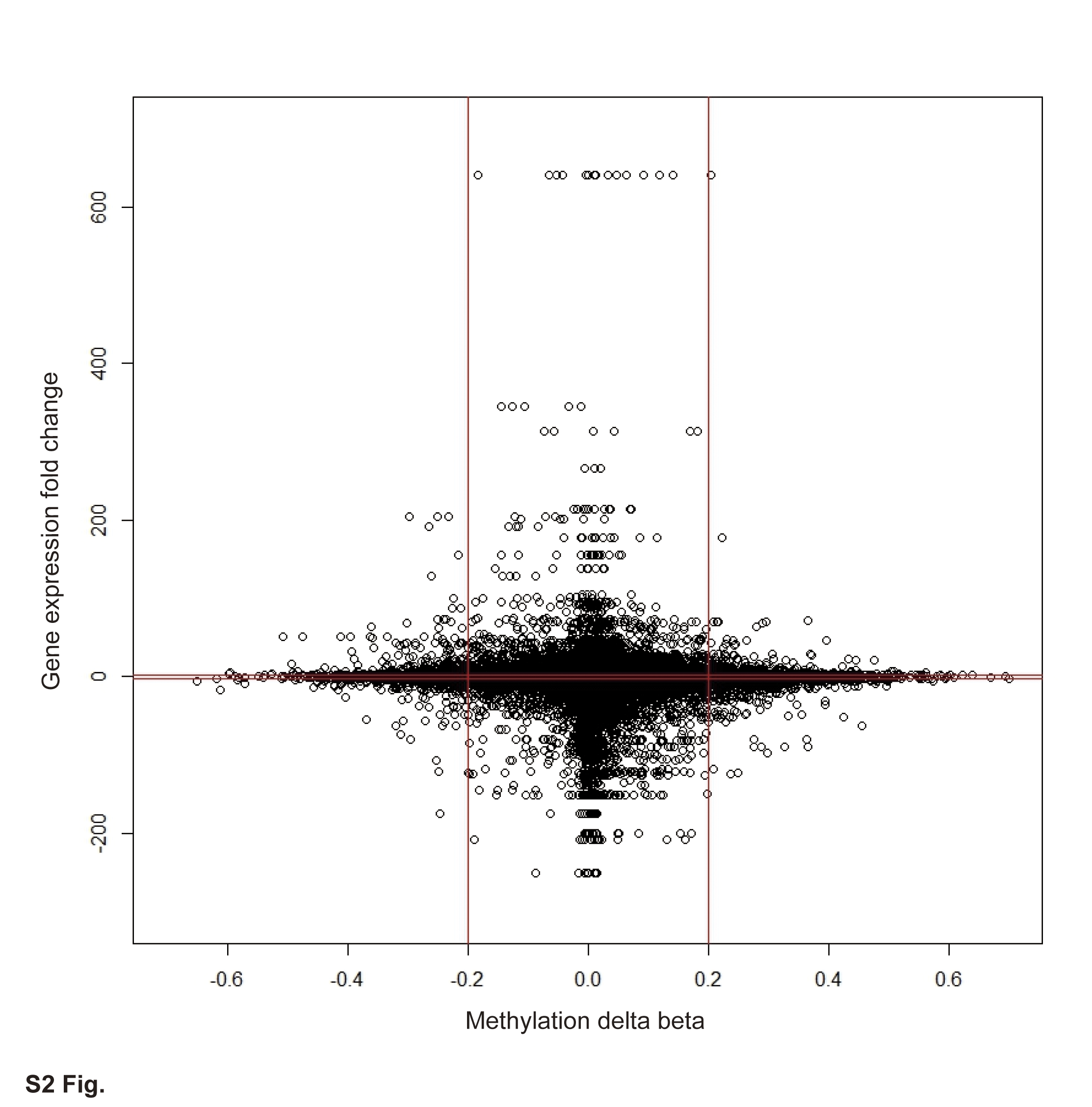

Supplement: S2 Fig — Each plot represents the values obtained from a single gene using integrated analyses. DNA methylation β values and gene expression levels are plotted along the abscissa and the ordinate, respectively. Red lines show the cut-off border. For methylation, Δβ for hypermethylation is > = 0.2, Δβ for hypomethylation is = < −0.2. For gene expression, the cut-off for increased/decreased expression was a ±2-fold change. (TIF) [file pone.0171431.s002.tif]
